# Prevalence of Psychosocial and Behavioral Aspects in Victims of Motorcycle Accidents in Civil Hospital, Karachi

**DOI:** 10.7759/cureus.4473

**Published:** 2019-04-16

**Authors:** Syeda Batool Zehra, Dua Fatima, Aleena Fatima Haider, Maratib Ali

**Affiliations:** 1 Orthopaedics, Civil Hospital Karachi, Dow Medical College, Karachi, PAK; 2 Internal Medicine, Civil Hospital Karachi, Dow Medical College, Karachi, PAK; 3 Orthopaedics, Dr. Ruth KM Pfau Hospital, Karachi, PAK

**Keywords:** behavioral aspects, motorcycle accidents, psychosocial aspects

## Abstract

Introduction

Motorcycles are common in a developing nation like Pakistan. In addition to their benefits, motorcycles carry a high risk for accident and injury. Many factors can exacerbate the risk of motorcycle operation including the use of mobile phones while riding, a lack of knowledge of traffic rules, not following road rules or non-satisfactory vehicle health and road conditions. Along with these physical factors, some psychosocial aspects also impact risks, including aggressive behavior of drivers or variations in driving patterns associated with changes in driver mood.

Objective

We conducted this study to determine the association of rider/operator behavioral and psychosocial factors with motor vehicle crashes.

Methods

We conducted a cross-sectional study on the patients of Civil Hospital in Karachi, Pakistan aged between 15 to 65 years. Data were collected from 150 patients in the outpatient department and emergency room via a questionnaire assessing driver biodata, license details, socioeconomic status, and their behavioral and psychosocial conditions. Inclusion criteria were limited to patients with motorcycle accidents only; patients involved in accidents from any other form of vehicle were excluded.

Result

Of the 150 patients, 70% were reported varying their driving speed with fluctuating moods, 80% rode aggressively when they have any social or financial issue, and 57% became annoyed with another driver’s behavior-all of which highlight the influence of psychosocial factors in motorcycle crashes. Concerning behavioral factors, 88% of drivers were involved in unofficial races, 44% reported overtaking slower drivers, and 80% violated traffic signals on a regular basis. These results suggest that behavioral and psychosocial factors have a major influence on the victims of motorcycle accidents and are an important cause of injury due to crashes.

Conclusion

Psychosocial and behavior aspects play a critical role in motorcycle accidents. Riders experiencing family-related or social-related stress and those with an aggressive personality are more prone to have a motorcycle accident than riders who do not have those stressors. Additional measures should be taken to raise awareness regarding these important contributing factors, including stress management in driving education.

## Introduction

Road traffic accidents pose a significant hazard to the lives of individuals worldwide. According to the World Health Organization, without sustained action, road traffic crashes are predicted to become the seventh leading cause of death by 2030 [1]. Motor vehicle collisions are an important yet preventable cause of morbidity and mortality in the developing world. Approximately 10 million crashes occur annually worldwide, and of those crashes that result in fatalities, nearly three-quarters of the deaths occur in developing countries [2]. Risk factors leading to motor vehicle accidents include but are not limited to environmental, social, psychological, and behavioral causes. However, psychosocial and behavioral issues are an essential cause of the increasing rate of motor vehicle collisions. The word psychosocial is defined as “relating to the interrelation of social factors and individual’s thought and behavior,” whereas behavior refers to “the way humans act and interact.”

In Pakistan, police report a gradual increase in the number of motor vehicle collisions, injuries, and deaths in the public sector since 1956 [2]. The death rate from road traffic accidents is four per 100,000 people and 15 per 10,000 vehicles in Pakistan [3]. Non-fatal injury rates of 19 per 10,000 people have been reported in Karachi alone [4]. Various types of motor vehicles are being used in Pakistan, including cars, buses, rickshaws, cycles, and motorcycles. Accidents due to motorcycle riding have different attributes as compared to other vehicles. Motorcyclists usually ride at high speeds, pass more, and move within small gaps in congested traffic as compared to other motor vehicles [5]. In recent years, higher rates of injuries and death among motorcycle riders have been reported due to increasing use [6]. The causes of increasing road traffic accidents are growing, including lack of enforcement of traffic safety rules, an increase in the number of vehicles, poor quality of roads, and inappropriate public health infrastructure. However, an individual rider's behavior, psychosocial condition, and driving practices are also key contributors to road traffic accidents [7].

While road traffic accidents pose a hazard to the public, they, unfortunately, remain largely unstudied around the globe. Studies have been done in the context of risk factors and causes of road traffic accidents; however, underlying psychosocial and behavioral aspects remain open for further research. The aim of our study is thus to focus on these aspects of motorcycle accidents to help mitigate future accidents.

## Materials and methods

We conducted a cross-sectional study from July 2018 to November 2018, collecting data primarily from the Civil Hospital in Karachi, Pakistan. Cross-sectional data of 150 male victims of motorcycle accidents were obtained from the inpatient and the outpatient departments of the hospital. Initially, a questionnaire was administered, consisting of fields collecting personal details of the male subjects, including biodata, socioeconomic status, license details, the condition of the motorcycle before the accident, and any previous history of road traffic accidents. Special emphasis was given to determine the subject's psychosocial and behavioral characteristics by inquiring about his motorcycle riding skills.

This questionnaire was translated in Urdu to make it understandable for the subjects and was administered directly to them after they provided oral consent to prevent any chance of ambiguity. Data collected were then entered and analyzed through IBM SPSS Statistics for Windows, Version 20.0 (IBM Corp., Armonk, NY, USA). Descriptive statistics were obtained and reported in percentages. A univariate level analysis was also performed to identify the association between different variables in the data. Inclusion criteria included male patients presenting after a motorcycle accident in Civil Hospital, Karachi.

## Results

We collected data from 150 motorcycle accident victims, of whom 31% were younger than 18 years, 50.7% were aged between 19 and 35 years, and 18.3% were age 36 to 65 years. Since most study participants were younger than 18 years, they did not have a license and were unaware of the basic traffic rules. Most (73%) crash victims reported they did not have a license while 18% had a permanent license, and 10% had a learner license.

Regarding the breaking of road rules, 85% of our study participants reported breaking road rules at least once a month, 8% three times per month, and 7% once per week (four times per month). Regarding helmet use, notably, 59% did not wear a helmet at all, 11% seldom wore a helmet, 6% wore a helmet frequently, and only 24% people reported always wearing a helmet. Regarding reported average speed, 44% stated they drove fast, 41% drove according to the condition of traffic, and only 15% drove slow.

Table 1 presents the psychosocial behavior of the victims. Notably, 80% of people always rode aggressively when they had any social or financial problem. Seventy-two percent reported their speed always depended on their moods. In addition, 44% of riders always sounded their horn to indicate their annoyance to another driver, 16% sometimes got annoyed, and 39% were never annoyed. Fifty-seven percent of riders agreed that they always got angry due to another driver’s behavior.

**Table 1:** Psychosocial aspects influencing riding

	Never	Sometimes	Always
	%	%	%
Get angered by another driver's behavior	33.8	9.2	57.0
Sound horn to indicate annoyance to another driver	39.4	16.2	44.4
Speed depends on my mood	16.9	11.3	71.8
Ride aggressively when have social or financial problems in my family	9.9	9.9	80.3

Table 2 represents the errors in riding among the male victims of motorcycle accidents. Approximately 80% reported they always underestimated the speed of an upcoming vehicle. Thirty-eight percent reported always applying sudden brakes, and 52.8% never applied sudden brakes. It is also worth mentioning that 77.5% of riders did not have side mirrors in their motorcycles and only 22.5% had side mirrors. Of this 22.5% of riders, 58% always failed to check their motorcycle mirrors before pulling out or changing lanes. Forty-one percent said that they always nearly hit a vehicle when turning left or right, while 58% have never been through this. Sixty-four percent reported they always failed to notice pedestrians when turning into a side street. Ninety-seven percent of riders rested when they got tired while riding, while 91% reported feeling distracted when riding.

**Table 2:** Riding errors

	Never	Sometimes	Always
	%	%	%
Fail to notice pedestrians while turning into a side street	35.9	0.0	64.1
Collide with a vehicle from inside while turning left or right	57.7	1.4	40.8
Fail to check mirror before pulling out or changing lanes	23.2	19.0	57.7
Underestimate the speed of oncoming vehicle when passing	19.0	1.4	79.6
Apply sudden brakes	52.8	9.2	38.0

Table 3 shows the traffic violations committed by the study participants. Of special note, 88% of riders reported they always got involved in unofficial races. Forty-four percent of riders reported they always became impatient, and 46% never became impatient with a slow driver and passed them smoothly. Fifty percent reported that they always disregarded speed limits at night or early mornings. Eighty percent reported always crossing a junction knowing the traffic lights had already turned red. Seventy percent of the subjects reported they always rode especially close to other vehicles on the road.

**Table 3:** Behavioral violation of traffic rules

	Never	Sometimes	Always
	%	%	%
Ride close to the vehicle	23.9	5.6	70.4
Cross junctions knowing the lights have turned red	18.3	1.4	80.3
Disregard speed limits early mornings and late night	36.6	13.4	50.0
Pass slow drivers	46.5	9.9	43.7
Become involved in unofficial races with other riders	10.6	1.4	88.0

## Discussion

The world is becoming a global village with different ways of communication among the masses. Different modes of transportation play a vital role in developing links among the communities. Road transportation includes buses, cars, cycles, and motorcycles. As motorcycles provide a faster commute in a cost-effective, fuel-efficient way, they have become the most common means of transportation in developing countries [8]. Along with these benefits, motorcycles carry a great health hazard as motorcycle crash injuries can prove devastating. As a developing nation, Pakistan is experiencing increasing numbers of motorcycle accidents daily, resulting in the need to explore the social, environmental, behavioral, and psychological causes of the increasing rate of accidents.

According to one study, the use of mobile phones while riding results in a four-fold increase in the risk of motor vehicle accidents [2]. Approximately 85% of accident victims reported using mobile phones while riding. Helmet use is one of the most important safety measures for motorcycle riders, and non-usage can lead to severe injuries. According to another study, rider compliance with helmet use differs based on geographic area. For example, in urban Indonesia, about 89% of riders reported wearing a helmet; in Karachi, Pakistan, 56% of riders reported wearing a helmet, while in Vietnam, only 30% of riders reported wearing a helmet [9]. In our study, only 41% of crash victims reported wearing a helmet while riding. We also found that 85% of riders broke road rules at least once a month, which is an important factor leading to the increase in accidents in Karachi. A study conducted on motorcycle accidents in China revealed that about 45% of motorcycle accidents occur among riders who do not follow traffic rules [10].

High speeds and readiness to violate traffic rules result in a higher incidence of accident risk [11]. In our study, we found that 41% of victims consistently drove fast. Australian research concludes that the feelings and sentiments of the riders play a pivotal role in influencing their intentions and behavior and should always be considered when it comes to safe riding (Doctoral dissertation: Tunnicliff DJ. Psychosocial Factors Contributing to Motorcyclists' Intended Riding Style: An Application of an Extended Version of the Theory of Planned Behaviour; Queensland University of Technology; 2006). Our study concurs, indicating that 72% of riders say their speed depended on their mood. Eighty percent of subjects indicated they rode aggressively when they were suffering from any social or financial issue, 57% of them became angered by another driver’s behavior, and 44% of them sounded their horn to indicate annoyance to another driver. Breaking road rules intentionally or unintentionally will ultimately affect the number of accidents worldwide. A study shows that young male drivers are more likely to break traffic rules [12]. In our study, we focused more on the behavioral, psychological, and social aspects of traffic violations and errors. Eighty percent of riders underestimated the speed of an oncoming vehicle when passing, and 58% failed to notice pedestrians, resulting in accidents. Seventy percent rode especially close to other vehicles, while 44% became impatient with a slow driver and passed them. Approximately 88% of riders said that they became involved with unofficial races with other riders, which is a very important psychosocial factor of motorcycle accidents.

Our study was limited in that it was a hospital-based study and included only Civil Hospital, Karachi. Only men were included since generally women do not ride motorcycles in Karachi. Pedestrian victims and victims of non-motorcycle-induced accidents were excluded from this study. Due to our small sample size, our study may not reflect the full spectrum of motorcycle accidents and their causes.

## Conclusions

Psychosocial and behavior aspects play a major role in motorcycle accidents, and riders experiencing family or social problems and riders who have an aggressive personality are more prone to have motorcycle accidents. Measures should be taken to raise awareness regarding these important contributing factors. Riders should be evaluated for stress management skills during their driving tests, and special emphasis should be given to improving awareness of psychosocial and behavior aspects of riders to reduce the rates of motorcycle accidents.

## References

[1] (2019). World report on road traffic injury prevention. Geneva: World Health Organization.

[2] Hyder AA, Ghaffar A, Masood TI (2000). Motor vehicle crashes in Pakistan: the emerging epidemic. Inj Prev.

[3] Rahman AF (2004). The burden of road traffic injuries in South Asia: a commentary. J Coll Physicians Surg Pak.

[4] Razzak JA, Luby SP (1998). Estimating deaths and injuries due to road traffic accidents in Karachi, Pakistan, through the capture-recapture method. Int J Epidemiol.

[5] Horswill MS, Helman S (2003). A behavioral comparison between motorcyclists and a matched group of non-motorcycling car drivers: factors influencing accident risk. Accid Anal Prev.

[6] Clarke DD, Ward P, Bartle C, Truman W (2019). In depth study of motorcycle accidents. Department for Transport.

[7] Ali SA, Kanpurwala MA, Aslam M, Siddiqui S, Khan S, Haider S, Tahir M (2015). Association of motorbike accidents with behavior related factors in Karachi. J Stud Manage Plan.

[8] Sai Praveen V, Ray GG (2015). A study on motorcycle usage and comfort in urban India. Proceedings of the 19th Triennial Congress of the IEA, Melbourne.

[9] Khan I, Khan A, Aziz F, Islam M, Shafqat S (2008). Factors associated with helmet use among motorcycle users in Karachi, Pakistan. Acad Emerg Med.

[10] Li Y, Qiu J, Liu GD (2008). Motorcycle accidents in China. Chin J Traumatol.

[11] Elander J, West R, French D (1993). Behavioral correlates of individual differences in road-traffic crash risk: an examination of methods and findings. Psychol Bull.

[12] Yagil D (1998). Gender and age-related differences in attitudes toward traffic laws and traffic violations. Transport Res F-Traf.

